# Substitution of outpatient care with primary care: a feasibility study on the experiences among general practitioners, medical specialists and patients

**DOI:** 10.1186/s12875-016-0498-8

**Published:** 2016-08-09

**Authors:** Sofie J. M. van Hoof, Marieke D. Spreeuwenberg, Mariëlle E. A. L. Kroese, Jessie Steevens, Ronald J. Meerlo, Monique M. H. Hanraets, Dirk Ruwaard

**Affiliations:** 1 Department of Health Services Research, Faculty of Health Medicine and Life Sciences, Maastricht University, P.O. Box 616, 6200 MD Maastricht, The Netherlands; 2 Research Centre for Technology in Care, Zuyd University of Applied Sciences, Heerlen, The Netherlands; 3 Department of Health Services Research, Faculty of Health, Medicine and Life Sciences, Maastricht University, Maastricht, The Netherlands; 4 Primary Care Organisation Care In Development ZIO, Wilhelminasingel 81, 6221 BG Maastricht, The Netherlands; 5 Department of Patient and Care, Academic Hospital Maastricht azM, P.O. Box 5800, 6202 AZ Maastricht, The Netherlands

**Keywords:** Collaboration, Substitution, Primary care, Outpatient care

## Abstract

**Background:**

Reinforcing the gatekeeping role of general practitioners (GPs) by embedding specialist knowledge into primary care is seen as a possibility for stimulating a more sustainable healthcare system and avoiding unnecessary referrals to outpatient care. An intervention called Primary Care Plus (PC+) was developed to achieve these goals. The objective of this study is to gain insight into: (1) the content and added value of PC+ consultations according to stakeholders, and (2) patient satisfaction with PC+ compared to outpatient care.

**Methods:**

A feasibility study was conducted in the southern part of the Netherlands between April 2013 and January 2014. Data was collected using GP, medical specialist and patient questionnaires. Patient characteristics and medical specialty data were collected through the data system of a GP referral department.

**Results:**

GPs indicated that they would have referred 85.4 % of their PC+ patients to outpatient care in the hypothetical case that PC+ was not available. Medical specialists indicated that about one fifth of the patients needed follow-up in outpatient care and 75.9 % of the consultations were of added value to patient care. The patient satisfaction results appear to be in favour of PC+.

**Conclusion:**

PC+ seems to be a feasible intervention to be implemented on a larger scale, because it has the potential to prevent unnecessary hospital referrals. PC+ will be evaluated on a larger scale regarding the effects on health outcomes, quality of care and costs (Triple Aim principle).

## Background

Rising healthcare expenditures in Europe are a threat to the financial stability and accessibility of health care [1]. As hospital care counts for the largest part of healthcare costs, reinforcing the gatekeeping role of the general practitioner (GP) to stimulate a shift from outpatient care to the less expensive setting of primary care may decrease healthcare spending by avoiding unnecessary outpatient care [2–4]. International literature shows that in cases in which GPs are more certain of diagnosis and/or treatment, they refer patients to outpatient care less frequently [5]. The problems GPs experience in managing uncertainties in diagnosis and treatment are considered to be partly caused by the fragmentation of healthcare systems and lack of communication with other healthcare providers [6–8]. Therefore, reinforcing the interaction between GPs and medical specialists and embedding specialist knowledge into primary care has been suggested to counter the strong existing fragmentation of primary care and outpatient care [9].

One example of an intervention in which specialist knowledge is embedded into primary care is performing care in outreach services. Previous studies have shown that integrating outreach services of specialist care into primary care could lead to a higher satisfaction of GPs with working processes, with no increase of total costs and a decrease of referrals to outpatient departments and specialist centres [10–15]. Other research on joint consultation interventions where medical specialists together with GPs examined and diagnosed patients in a primary care setting also showed a decrease in referrals to outpatient care [16, 17]. However, care in outreach clinics could also lead to increased healthcare costs. These were primarly caused by higher National Health Service (NHS) costs, overhead costs, medical and nurses staffing costs, travel time and costs and inefficient use of medical specialists’ time [13, 18]. Besides, other literature states that more medical specialist input could lead to immoderate medical consumption and over-diagnosis [19].

As part of a national policy to build a more sustainable healthcare system, the Dutch Ministry of Health, Welfare and Sport appointed nine regions across the Netherlands as pioneer sites, in April 2013. These pioneer sites are able to experiment with (new) interventions to accomplish the Triple Aim principle: reduced care costs per capita, along with improved population health and patient experiences [20]. An incentive for the pioneer sites to accomplish substitution is that the Dutch Ministry of Health, Welfare and Sport, healthcare organisations, health insurance companies and patient organisations have agreed that the volume growth for hospital care should be limited to 1.5 % in 2014 and only 1 % per year from 2015 to 2017. Moreover, primary care is allowed to grow by 1 % in 2014 and 1.5 % per year from 2015 to 2017 if they are able to establish that they contribute to substitution. The agreement also states that healthcare organisations and stakeholders in the Netherlands have a societal obligation to accomplish a decrease in healthcare costs [21].

One of these pioneer sites started to experiment with an intervention called Primary Care Plus (PC+). PC+ was developed to strengthen the cooperation between GPs and medical specialists and to strengthen the role of the GP as gatekeeper. The general aim of PC+ is to support GPs in treating patients by integrating specialist knowledge into primary care, aiming at fewer (unnecessary) referrals to outpatient care, without losing sight of the nature of primary care. In this feasibility study PC+ is assumed to accomplish a substitution effect by permitting medical specialists to perform short consultations in a primary care setting (GP practices) without the facilities of the hospital, and advising GPs about further treatment afterwards, while GPs retain their gatekeeping role. The idea is that medical specialists experience a barrier in running all kinds of tests in PC+, because they do not have the facilities, and learn to analyse a patient’s medical complaint with the generalist approach of a GP but with the expertise and experience of their specialised medical field.

Several hypotheses are presumed about how PC+ could contribute to the Triple Aim. Firstly, to accomplish reduced care costs PC+ is assumed to contribute to a decreased number of unnecessary referrals to outpatient care. Some patients, who before PC+ existed were referred to outpatient care, will now be referred to PC+. With the medical specialist’s advice to the GP after the PC+ consultation (and the improved communication between GPs and medical specialists), a GP will be better able to determine whether a referral to outpatient care is necessary [11, 14]. As a result, the total number of outpatient care referrals will decrease and overuse and misuse of outpatient care will be avoided. Together with the fact that outpatient care is more expensive (because of more overhead costs) than PC+, PC+ will result in a decrease in care costs.

Secondly, by avoiding unnecessary referrals and the possibility of overuse with the risk of adverse effects, the health of the population is assumed to improve or at least remain the same [12, 14]. Patients that really need specialised outpatient care after a consultation in PC+ will still be referred to outpatient care.

Finally, PC+ is assumed to lead to improved patient experience of care, because patients can receive their care close to where they live, as well as in a timely and patient-centred way due to the GP’s coordinating role as gatekeeper of the healthcare system (12, 14).

A qualitative feasibility study at this pioneer site revealed that GPs and medical specialists believe PC+ could become a feasible intervention when the following conditions are met: (1) the project management should make arrangements on a governmental level; (2) the project management should arrange a collective integrated IT-system; (3) the project management together with involved GPs and medical specialists should determine the appropriate profile for medical specialists; (4) the project management together with involved GPs and medical specialists should design a referral protocol for eligible patients; (5) the project management should arrange deliberation possibilities for GPs and medical specialists, and (6) the project management together with involved GPs and medical specialists should formulate a diagnostic protocol [22]. According to the Medical Research Council (MRC) complex interventions framework it is important to test procedures of a new intervention in a feasibility or pilot phase before implementing it on a larger scale [23]. To answer the question of whether PC+ in its current form is feasible, a feasibility study was conducted in this pioneer site where medical specialists performed consultations in a primary care setting. It follows that one needs to know the content of PC+ consultations and the added value of PC+ according to its users (GPs, medical specialists and patients). The objectives of this study were to gain insight into the content and added value of PC+ consultations according to involved GPs and medical specialists, and to gain insight into patient satisfaction with PC+ as compared to patient satisfaction with outpatient care. Depending on the results of this study, the process and content of PC+ can be adapted before implementing PC+ on a larger scale.

## Methods

### Study design and setting

This feasibility study was conducted in the southern part of the Netherlands in the Maastricht-Heuvelland region at pioneer site ‘Blue Care’ between April 2013 and January 2014. This ‘Blue Care’ initiative is a partnership between the only academic hospital in Maastricht, the only primary care organisation in Maastricht-Heuvelland ‘Care in Development’ (in Dutch ‘Zorg in Ontwikkeling’), the patient representative foundation ‘House of Care’ (in Dutch ‘Huis voor de Zorg’), and the most dominant health insurance company, VGZ, in this region. The term Blue Care was conceived as an analogy for green power in an effort to designate the importance of behavioural change to achieve more sustainable health care [24].

Patients were informed about the study and asked to give their consent to participate when making an appointment for the consultation with the medical specialist in PC+ or in outpatient care. Only patients that provided informed consent were included in this study.

### Intervention

The Blue Care region covers 175,000 inhabitants who are all registered with one of the 82 GPs in one of the 57 GP practices of ‘Care in Development’. During the PC+ feasibility study 17 GPs with 32,322 patients, working under the umbrella of primary care organisation ‘Care in Development’, were able to refer patients to PC+ in case they were uncertain of the diagnosis, treatment or necessity to refer a patient to outpatient care. Only these GPs participated in this PC+ feasibility study because they were prepared and willing to try out this new intervention. In six GP practices medical specialists performed PC+ consultations on a weekly or biweekly basis. In six other practices the GP referred their patients to the six PC+ practices. The intervention group included patients who received a PC+ consultation from the GPs of these twelve practices. The usual care group consisted of patients from these twelve practices who received a referral for outpatient care. Outpatient care is defined as usual care in hospitals. After a patient received a referral for PC+ or outpatient care, the patient called the GPs referral department of ‘Care in Development’, named TIPP (Transmural Interactive Patient Platform), for an appointment.

Medical specialists of the academic hospital Maastricht of five medical specialties (internal medicine, orthopaedics, dermatology, neurology and cardiology) performed short consultations (maximum of 20 min) in GP practices. The duration of a first outpatient consultation in the academic hospital Maastricht lasts 30–45 min. These medical specialists only had access to (diagnostic) materials that were available in GP practices, and thus were only able to perform care that did not require hospital facilities. Afterwards they provided the GP with advice on diagnosis, treatment or necessity to refer the patient to outpatient care. During the process of PC+, GPs remained responsible for the patient.

### Measurements

Patient characteristics (age and gender) and data about the medical specialty to which the patient was referred were collected through the TIPP data system. Data regarding the reasons for GPs to refer patients to PC+, the content and added value of the PC+ consultations according to medical specialists, and the satisfaction of patients with PC+ compared to outpatient care were collected through three questionnaires completed by GPs, medical specialists and patients, respectively. GPs and medical specialists only completed questionnaires for patients who received a referral for PC+. The patient satisfaction survey was conducted among patients who received a referral for PC+ as well as among patients who received a referral for outpatient care.

### Questionnaire GP

GPs completed a short questionnaire immediately after referring a patient to PC+, including questions about: (1) the duration of the patient’s complaints; (2) the reason for referring the patient to PC+ (multiple answers could be given), and; (3) the alternative choice of the GP in the hypothetical case PC+ was not available.

### Questionnaire medical specialist

Medical specialists completed a short questionnaire immediately after the PC+ consultation. Topics were: (1) the duration of the consultation; (2) the actions completed by the medical specialist during the consultation; (3) the advice of the medical specialist to the referring GP regarding follow-up, and; (4) the added value of the consultation according to the medical specialist.

In case of non-response, GPs and medical specialists received up to two reminders within four weeks.

### Questionnaire patients

To investigate patient satisfaction with PC+, we compared the PC+ patients with non-acute patients who were directly referred by their GP via TIPP to the outpatient department of the involved medical specialties in a hospital. Patients were asked to participate in a digital patient satisfaction survey when they called to schedule an appointment with TIPP. They received the survey within seven days of the completion of their consultation with the medical specialist in PC+ or in the hospital. The patient satisfaction questionnaire was developed by TIPP and was based on the Consumer Quality index [25]. The questionnaire included questions about marks (on a scale of 0-10) for: (1) the information given by the medical specialist; (2) the cooperation between the medical specialist and the employees of the institution (PC+ or hospital); (3) the result of the treatment; (4) the overall score of the medical specialist and; (5) the institution (PC+ or hospital). After the consultation, non-responders received up to two reminders in a six-week period.

### Statistical analysis

Data are described using absolute counts and percentages for categorical variables; means and standard deviations are used for continuous variables. Differences between characteristics and satisfaction of patients referred to PC+ and outpatient care were analysed using Pearson Chi-Square tests for categorical variables and independent t-tests for continuous variables. Normality was checked using the visual inspection of the Q-Q plot. If variables were not normally distributed, non-parametric tests were used. *p-*values less than 0.05 were considered statistically significant. Analyses were performed using IBM SPSS Statistics 21 (SPSS Inc., Chicago, IL).

## Results

### Participants

**Fig. 1:**
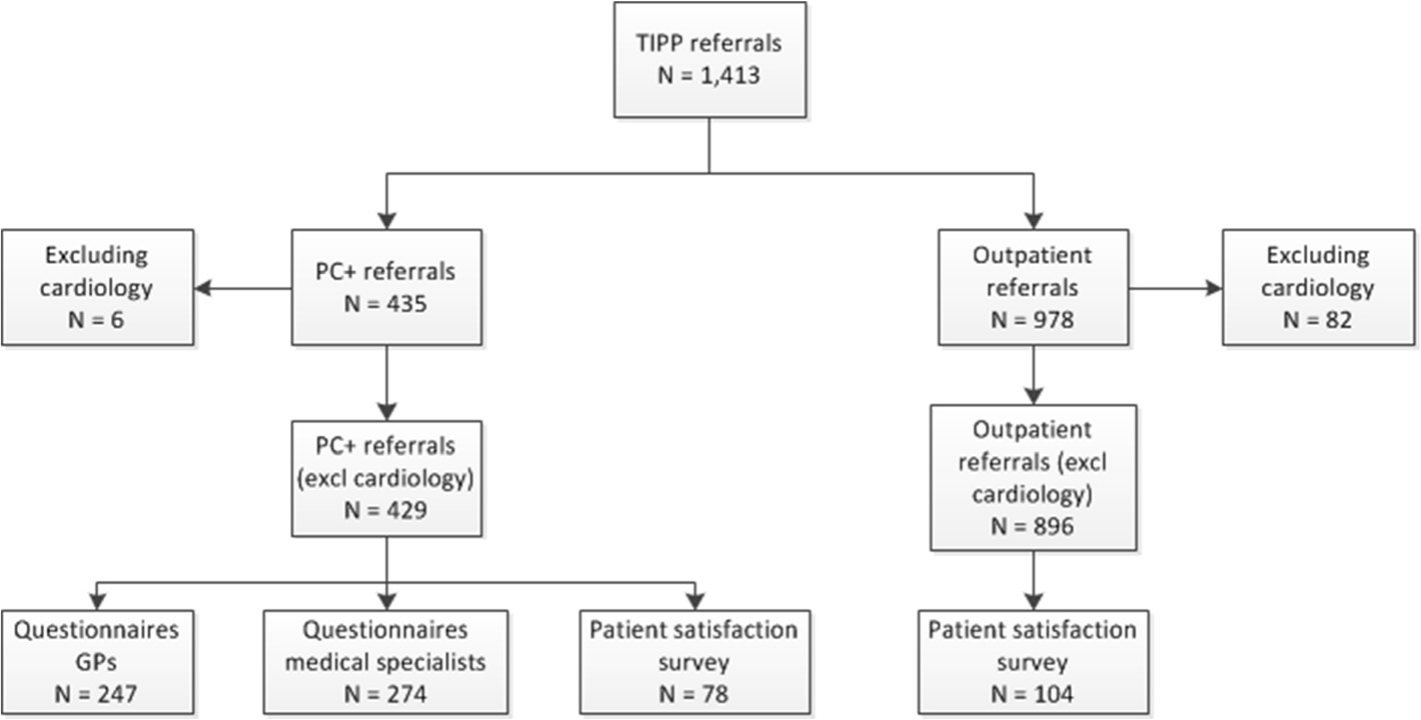
Sampling flow diagram

During the feasibility study 1,413 patients were referred to PC+ or directly to outpatient care in one of the involved medical specialties. Cardiology only received 6 referrals to PC+ before deciding to stop the intervention. The cardiology department of the academic hospital Maastricht decided to stop the PC+ intervention because they indicated that they needed more diagnostic equipment (e.g., an ultrasound device) to be more certain of the diagnosis and treatment. Therefore, the cardiology referrals to PC+ and outpatient care were excluded from further analysis. Of the remaining 1,325 patients 429 patients were referred to PC+ and 896 patients were referred directly to outpatient care via TIPP. Of the 429 PC+ referrals, 102 referrals were to internal medicine, 85 to neurology, 115 to orthopaedics, and 127 to dermatology. Response rates of the GP and medical specialist questionnaires were 58 % and 64 %, respectively. Response rates for the patient satisfaction questionnaires were 18 % for the PC+ patients and 12 % for the patients who received outpatient care (See Fig. 1 and Table 1.)

**Table 1:** Characteristics and number of referrals per medical specialty (to PC+ or outpatient care)

	PC+	Outpatient care	Overall	*P*-Value
	(*N* = 429)	(*N* = 896)	(*N* = 1325)	
Age in years (mean ± SD)	54.7 ± 17.0	53.8 ± 16.8	54.1 ± 16.9	0.333
Gender				
Male % (*N*)	41.5 (178)	38.6 (346)	39.5 (524)	0.316
Female % (*N*)	58.5 (251)	61.4 (550)	60.5 (801)	
Medical specialty				
Internal medicine % (*N*)	23.8 (102)	16.9 (151)	19.1 (253)	
Neurology % (*N*)	19.8 (85)	21.8 (195)	21.1 (280)	
Orthopaedics % (*N*)	26.8 (115)	31.6 (283)	30.0 (398)	
Dermatology % (*N*)	29.6 (127)	29.8 (267)	29.8 (394)	

The groups of patients referred to PC+ and outpatient care did not differ in age and gender (*p* = 0.333 and *p* = 0.316, respectively; see Table 1).

### Results of the GP questionnaire

The mean duration of complaints for patients referred to PC+ was 47.7 weeks (SD 52.7) (see Table 2). Patients with orthopaedic medical complaints experienced the longest mean duration of the complaint, specifically 51.6 weeks (SD 53.3). Patients with medical complaints related to internal medicine experienced the shortest mean duration of the complaint at the time of the referral by the GP, namely 40.9 weeks (SD 44.2).

**Table 2:** Results of the GP questionnaire

	Overall	Internal medicine	Neurology	Orthopaedics	Dermatology
	(*N* = 247)	(*N* = 56)	(*N* = 48)	(*N* = 56)	(*N* = 87)
Duration of complaints in weeks (mean ± SD)	47.7 ± 52.7	40.9 ± 44.2	49.9 ± 61.7	51.6 ± 53.3	47.9 ± 52.5
Reasons for referring % (*N*)^a^					
To confirm disease	21.5 (53)	1.8 (1)	25.0 (12)	23.2 (13)	31.0 (27)
To exclude disease	17.4 (43)	26.8 (15)	20.8 (10)	10.7 (6)	13.8 (12)
Screening unclear pathology	43.3 (107)	60.7 (34)	37.5 (18)	42.9 (24)	35.6 (31)
Controlling known condition	5.7 (14)	3.6 (2)	0.0 (0)	5.4 (3)	10.3 (9)
Reassurance	8.1 (20)	7.1 (4)	16.7 (8)	5.4 (3)	5.7 (5)
Upon patient request	9.3 (23)	1.8 (1)	10.4 (5)	10.7 (6)	12.6 (11)
Upon medical specialist advice	0.8 (2)	3.6 (2)	0.0 (0)	0.0 (0)	0.0 (0)
Other	6.9 (17)	3.6 (2)	4.2 (2)	14.3 (8)	5.7 (5)
Choice GP in hypothetical case PC+ was not available % (*N*)					
Keep patient in primary care	11.3 (28)	16.1 (9)	10.4 (5)	8.9 (5)	10.3 (9)
Refer to outpatient care	85.4 (211)	82.1 (46)	87.5 (42)	87.5 (49)	85.1 (74)
Refer to other healthcare provider^b^	3.3 (8)	1.8 (1)	2.1 (1)	3.6 (2)	4.6 (4)

^a^More than one answer could be provided

^b^Referrals to other are specified as referrals to a physiotherapist, a dietician or to mental health care

GPs indicated several reasons for referring patients to PC+. Overall, GPs referred their patients most often to PC+ because they wanted the patients to be screened for an unclear pathology (43.3 %). Also ‘to confirm a disease’ or ‘to exclude a disease’ were frequently mentioned reasons for referring patients to PC+ (21.5 % and 17.4 %, respectively). Reasons for GPs to refer patients to PC+ differed per medical specialty. For internal medicine, the most frequently cited reason to refer was to screen for unclear pathology (60.7 %), while the least common reason was to confirm the disease (1.8 %). For the remaining medical specialties, the reasons to refer were consistent with the overall pattern.

Finally, GPs were asked what they would do in the hypothetical case PC+ was not available. Overall, in 85.4 % of cases the patient would have been referred to outpatient care in the case that PC+ was not available, with little differences per medical specialty.

### Results of the medical specialist questionnaire

The mean duration of the consultation in minutes differed significantly between the medical specialties (*p* ≤ 0.000) (see Table 3). Internists and neurologists had the longest consultations of 33.9 (SD 11.9) and 23.4 (SD 7.6) minutes, respectively, while dermatologists and orthopaedics needed the fewest consultation time of 13.1 (SD 3.9) and 15.9 min (SD 4.9), respectively.

**Table 3:** Results of the medical specialist questionnaire

	Overall	Internal medicine	Neurology	Orthopaedics	Dermatology
	(*N* = 274)	(*N* = 84)	(*N* = 64)	(*N* = 56)	(*N* = 70)
Duration of consultation in minutes (mean ± SD)	22.7 ± 11.8	33.9 ± 11.9	23.4 ± 7.6	15.9 ± 3.9	13.1 ± 4.9
Actions during consultation % (*N*)					
Limited medical history	39.8 (109)	20.2 (17)	60.9 (39)	16.1 (9)	62.9 (44)
Extensive medical history	56.6 (155)	76.2 (64)	34.4 (22)	82.1 (46)	32.9 (23)
Limited physical examination	47.1 (129)	25.0 (21)	59.4 (38)	37.5 (21)	70.0 (49)
Extensive physical examination	43.1 (118)	58.3 (49)	29.7 (19)	62.5 (35)	21.4 (15)
Giving information	25.2 (69)	33.3 (28)	35.9 (23)	17.9 (10)	11.4 (8)
Prescription medication	6.2 (17)	4.8 (4)	4.7 (3)	1.8 (1)	12.9 (9)
Small operation	0.4 (1)	0.0 (0)	0.0 (0)	0.0 (0)	1.4 (1)
Other	6.9 (19)	3.6 (3)	10.9 (7)	12.5 (7)	2.9 (2)
Follow-up after PC+ % (*N*)					
Complaint resolved	37.6 (103)	21.4 (18)	40.6 (26)	48.2 (27)	45.7 (32)
Referral back to GP	19.0 (52)	47.6 (40)	4.7 (3)	3.6 (2)	10.0 (7)
Extra consultation in PC+	21.5 (59)	29.8 (25)	34.4 (22)	5.4 (3)	12.9 (9)
Extra consultation in outpatient care	21.9 (60)	1.2 (1)	20.3 (13)	42.9 (24)	31.4 (22)
Added value of consultation? % (*N*)					
Yes	75.9 (208)	95.2 (80)	65.6 (42)	66.1 (37)	70.0 (49)
No, consultation GP would have been sufficient	9.5 (26)	2.4 (2)	26.6 (17)	3.6 (2)	7.1 (5)
No, direct referral hospital necessary	13.9 (38)	2.4 (2)	7.8 (5)	30.4 (17)	20.0 (14)
No, reason unknown	0.7 (2)	0.0 (0)	0.0 (0)	0.0 (0)	2.9 (2)

Overall, the consultation consisted primary of taking an extensive medical history (56.6 %), and/or a limited or extensive physical examination (47.1 % and 43.1 %, respectively) (see Table 3).

The advice for follow-up actions differed between the medical specialties. In particular, internists indicated that only 1.2 % of patients needed a referral to outpatient care after PC+. For the remaining internal medicine patients, the complaint was taken care of after the consultation in PC+ (21.4 %), 47.6 % needed an extra consultation with the GP, and 29.8 % needed an extra consultation in PC+. Particularly, orthopaedic patients (42.9 %) and dermatology patients (31.4 %) more frequently needed a referral to outpatient care after PC+ compared to internal medicine (1.2 %) and neurology (20.3 %) patients. Overall, in 21.9 % of the cases, a follow-up in outpatient care was necessary.

The extent to which the medical specialist experienced the consultation in PC+ as an added value varied per medical specialty. Internists indicated that in 95.2 % of the cases the PC+ consultation had added value. For neurologists, orthopaedics and dermatologists, this percentage varied between 65.6 and 70.0 %. In particular, orthopaedic patients needed a direct referral to outpatient care, instead of a consultation in PC+ (30.4 %).

### Results of the patient satisfaction questionnaire

PC+ responders to the patient satisfaction questionnaire were younger (50.2 years, SD 14.3) than the outpatient care responders (55.7 years, SD 15.1) (*p* = 0.013). PC+ non-responders (55.8 years, SD 17.4) were older than outpatient care non-responders (53.5 years, SD 17.0) (*p* = 0.043).

**Fig. 2:**
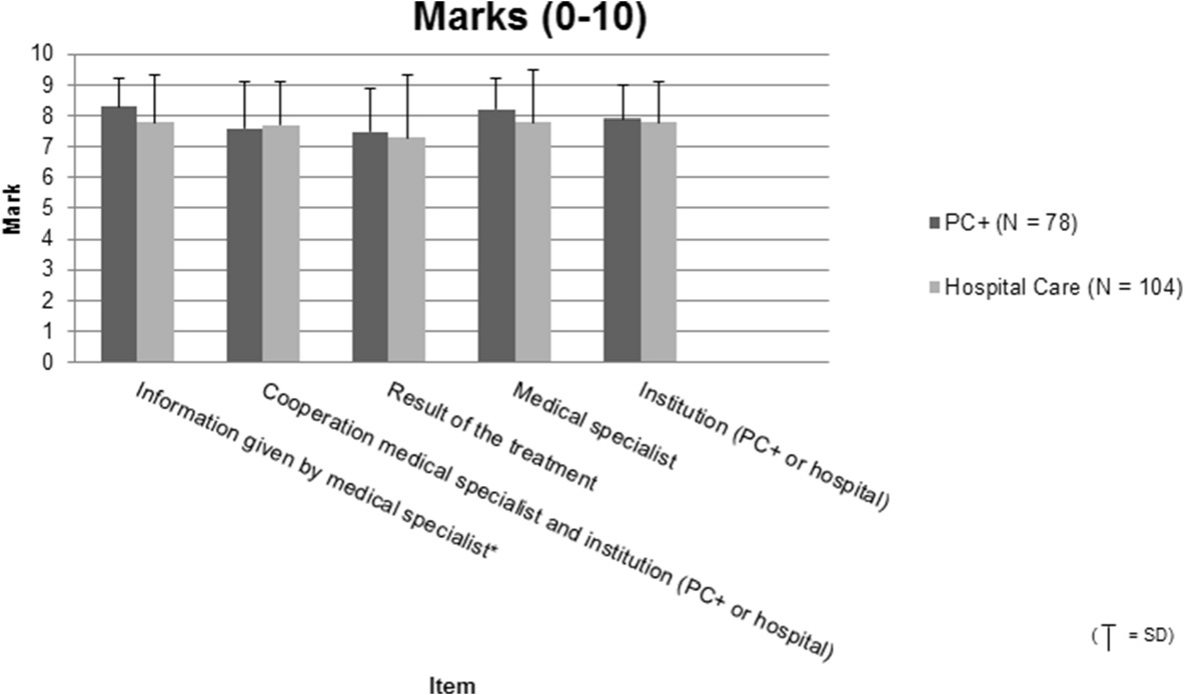
Results patient satisfaction questionnaire. Legend: ‘0’ is the worst possible mark; ‘10’ is the best possible mark; * Statistically significant, *P*-value <0.05

Regardless whether they were referred to PC+ or outpatient care, patient satisfaction results show a positive picture with average scores between 7.3 and 8.3. Only the results for the item ‘information given by the medical specialist’ indicated a significant favourable outcome of PC+ (8.3) as compared to the outpatient care group (7.8) (*p* = 0.006) (see Fig. 2). In general, the new PC+ intervention results in the same level of patient satisfaction as outpatient care.

The results are almost the same for all of the various medical specialties (data not shown).

## Discussion

The study objectives were to gain insight into the content and added value of PC+ consultations according to involved GPs and medical specialists, and to gain insight into patient satisfaction with PC+ compared to patient satisfaction with outpatient care. Results showed that GPs who referred their patients to PC+ would have, in most cases, referred their patients to outpatient care in the hypothetical case that PC+ was not available. Medical specialists indicated that only about one fifth of the patients needed follow-up in outpatient care after PC+. The patient questionnaire revealed high satisfaction rates for both PC+ and outpatient care and is significantly better in PC+ when taking the item ‘satisfaction with the information given by the medical specialist’ into consideration. To estimate whether PC+ is an intermediate station between primary and outpatient care or leads to substitution of outpatient with primary care, it is necessary to analyse ‘real’ referral figures.

The content and follow-up of PC+ depends to a large extent on the involved medical speciality. Previous qualitative research on the preconditions for PC+ showed that orthopaedists and dermatologists needed more outpatient care follow-ups because hospital facilities were necessary for diagnosis, such as X-ray facilities and biopsy materials, compared to the more contemplative specialty of internal medicine [22]. It seems logical that the demand of facilities differs between medical specialties because each specialty has a different patient population [26]. In addition, cardiology, in its current form, was not suitable for PC+. Cardiologists indicated that they needed more diagnostic facilities in PC+, such as an ultrasound device, to be more certain of the diagnosis and treatment. Moreover, cardiologists expect that an alternative substitution model, in which chronic cardiology patients have follow-ups in PC+ instead of in outpatient care, might work better [22]. It is therefore questionable if PC+, in its current singular form, is applicable to all kinds of medical specialties. In the next implementation phase, attention should be paid to the differences between medical specialties, their need for different resources in PC+, and different patient flows. A strength of this study is that it focused on analysing the feasibility of a substitution model in a population management setting across various medical specialties; other studies primarly focus on disease management programs and chronic care models [27–29]. In addition, the study involved experiences from multiple involved stakeholders (GP’s, medical specialists and patients), which is something that is seen as an important prerequisite in implementation research [30].

Although the questionnaires for GPs and medical specialists showed relatively high response rates, the response rate for the patient questionnaire was low. A comparable study on patient satisfaction rates with health care, measured with the Consumer Quality index survey in the Netherlands, showed a response rate of 33.1 % [25]. The patient questionnaire showed high levels of satisfaction for both PC+ and outpatient care. Patients seem to accept treatment by a medical specialist outside the context of a hospital. However, satisfaction levels tend to differentiate poorly and are not always an accurate representation of perceived quality of care [31, 32]. Furthermore, the group of patients referred to PC+ and the group of patients referred to usual care could not be compared on their medical needs and/or expectations. However, the results in this feasibility study were corrected for age and gender. In a larger observational study, confounding will be corrected for using propensity score matching on a range of measured baseline variables (including medical consumption).

The relatively small sample size and region specific characteristics (only one [academic] hospital and only one primary care organisation, and a long tradition of transmural collaboration) make it difficult to extrapolate the results of this study to other regions in the Netherlands and beyond.

According to the results of this study, PC+ seems to be a promising intervention to be implemented on a larger scale, as it could lead to the substitution of outpatient care with primary care and high patient satisfaction. Nevertheless, one should keep in mind that the cost-effectiveness of PC+ will depend on the tariff of a PC+ consultation, the extra overhead costs and the efficiency of the planning of medical specialists’ time [12, 13, 28]. Moreover, in future implementations different forms of PC+ and the creation of protocols for the various involved medical specialties should be considered.

## Conclusion

The conclusion of this study is that PC+ seems to be a feasible intervention to be implemented on a larger scale because it has the potential to prevent unnecessary referrals to outpatient care. The study resulted in a continuation and implementation of the PC+ project in the Blue Care region, including additional medical specialties. In addition, future research will also take into account retrospective and prospective referral data from other regions to discover trends with and without the existence of PC+.

## Abbreviations

GP, general practitioner; MRC, medical research council; NHS, national health service; PC+, primary care plus; TIPP, transmural interactive patient platform
